# Austin District Medical Society

**Published:** 1893-07

**Authors:** 


### Society Notes.

**The Austin District Medtcal Society** held its twenty-third quarterly session in Austin, June 22d ult. The attendance was fair,—larger than usual from adjacent country. In the absence of President Talley (of Temple), Dr. C. A. Danforth, of Granger, 1st Vice-President, presided. The program, as published in our last issue, was carried out. The paper, by Dr. W. J. Mathews, on "Treatment of Salpingitis without Operation," elicited interesting discussion, as did that of Dr. Cunningham on "Puerperal Eclampsia." Drs. C. O. Weller, A. N. Denton, G. W. Christian, Sec. Bennett and others participated, as did Dr. J. F. Y. Paine, of Galveston, Professor of Obstetrics in the Texas Medical College. Prof. Paine was made an honorary member; and Drs. H. B. Granberry, of Austin; E. M. Thomas, of Walburg; J. T. O'Bar, Ledbetter; J. L. Lee, Del Valle, C. J. Forbes, Round Rock, elected to active membership. The above mentioned papers, together with the substance of the remarks by the several gentlemen will appear in our next issue. Dr. Wallace and Dr. Cunningham being absent, their papers were not discussed.

Dr. Daniel read a paper, prepared by request, on the "So-called Bichloride of Gold Treatment; its Relation to the Profession and the People." [Not "a paper on the Keeley Cure, its Ethical Status," as announced in the program ; it is objected that the "Keeley Cure" *has* no ethical status.] The paper was very generally commended and by some members highly complimented, —the views held by the author being, in the main, endorsed. It elicited, however, some adverse criticism, and created considerable interest. We regret our inability to give this discussion. The paper will be published in this month's issue of the *Texas Sanitarian.*

